# Economic impact of inclusion of black soldier fly products in broiler diets: A comparison between conventional and higher animal welfare production systems in the Netherlands

**DOI:** 10.1016/j.psj.2024.104411

**Published:** 2024-10-15

**Authors:** Mark Leipertz, Henk Hogeveen, Helmut Saatkamp

**Affiliations:** Business Economics group, Wageningen University and Research, 6700 EW Wageningen, the Netherlands

**Keywords:** Black soldier fly, Economic, Insect-fed poultry production, Animal welfare production system, Protein replacement

## Abstract

The primary objectives of this study were to analyze economic feasibility of incorporating black soldier fly products in broiler feed and to compare the viability of alive (alive grown larvae (**AGL**)) or processed (defatted larvae meal (**DLM**) and insect oil) insect inclusion into the two Dutch broiler welfare systems: conventional and better life one star (**BLS**).

An existing simulation model for insect production was adapted to build an economic model for the insect-fed broiler breast meat supply chain. Amounts, full costs, and breakeven prices of different production setups were compared and the impacts of specific input parameters were tested by sensitivity and breakeven analyses.

Results indicated a hypothetical demand of 2.15 million tons wet matter of raw substrate to meet the total Dutch breast meat production (BLS broilers and 10% AGL inclusion). In case of 10% processed insect inclusion, the conventional broiler breast meat price increased from 3,947 to 6,069 €/t (53.7%) and the BLS breast meat price from 6,493 to 9,662 €/t (48.8%). Alive insect inclusion resulted in a smaller cost increase (42.2%) by skipping the costly processing step. To become price competitive, DLM or AGL must cost 618 or 801 €/ton dry matter (**tDM**), respectively, requiring a raw substrate mix price of −109 €/tDM.

High input amounts and costs currently hinder the widespread adoption of insect products in Dutch mass poultry feed. However, in the long run, using negative value substrates or targeting niche markets for broiler welfare with low AGL inclusion levels could become viable business models.

Research is needed on the impact of negative value substrates on insect production performance and contamination risks. Furthermore, the impact of varying AGL composition requires special attention.

## Introduction

An ever-increasing demand for high quality protein is fueled by the continuously growing world population in combination with rising incomes (OECD/FAO, 2020).

One important high quality protein source is poultry meat. Per capita and year, the Dutch citizens consume in average 22 kg poultry meat (Dagevos et al., 2022). This, plus export lead to a total yearly Dutch poultry meat production of approximately 1 Mio t broilers, which resemble approximately 200,000 t breast meat. Poultry meat intended for export is mostly reared in the conventional broiler welfare (**BW**) system which uses fast growing breeds. Induced by Dutch retailers, between 2014 and 2016 the market of fresh broiler meat that is sold in the Netherlands mostly changed to the higher BW system “New Dutch Retail Standard” that uses slower growing broilers (Saatkamp et al., 2019). However, due to the campaign of the largest Dutch animal welfare NGO (Dierenbescherming) the “Better life one star” (**BLS**) broiler production, which prescribes even more space and time to slaughter per broiler, recently became the new standard that is sold in Dutch supermarkets. Consequently, Berntsen et al. (2021) expect that in 2025 20–40% of the Dutch broiler production remains conventional for export, the New Dutch Retail Standard disappears from the marked, BLS production increases in importance with 60–80% market share, and organic production stays at a low level of 1–3%.

Despite being important for Dutch nutrition, poultry production faces the problem that soybean meal, the main protein supplier in the feed, is connected with deforestation and long transportation distances. Including insects in broiler feed could enable circular farming and enhance the sustainability of the supply chain. There are two possibilities to include insects in broiler feed: as alive insects fed additionally to commercial broiler feed (**CBF**) and as processed insect products incorporated in the CBF (Dörper et al., 2024). The alive inclusion of insects is expected to save costs as the (costly) processing step can be skipped (Leipertz et al., 2024), but that may also cause some drawbacks in the production process.

While there are papers available on the costs of insect production (Leipertz et al., 2024; Niyonsaba et al., 2021), the analysis of the competitiveness of insect products with other feed ingredients is scarce (Nunes et al., 2023; Tavares et al., 2022). Nunes et al. (2023), attest an advantageous 100% substitution of fish meal with black soldier fly meal up to a price of 2.65 USD/kg black soldier fly meal. However, their study did not take all aspects into account. Even if the returns of investment do not significantly differ up to a price of 2.65 USD/kg, it does not mean that the inclusion is beneficial. In fact, it is visible that the mean of the return of investment is always lower when black soldier fly meal, with a price between 2.00–3.50 €/kg, is included in the diet. Also, Tavares et al. (2022) use wrong calculation methods^1^ in their basic economic analysis of the viability of insect meal as broiler feed ingredient. The flaws in previous researches indicate a need for an in-depth and representative study into the viability of using insects as alternative protein source in animal production.

The objective of our research was to analyze and compare the viability of alive or processed insect inclusion in broiler feed for the two main BW systems: conventional and BLS. We especially focused on breakeven prices or other circumstances that have to be met to enter the (mass) poultry feed market.

The correctly applied full cost approach, the simulation of the whole insect-fed broiler breast meat supply chain at the same time and the inclusion of realistic input parameter variations distinguishes this study from previous research. It enabled the analysis of cross effects between insect and broiler breast meat production and allowed a thorough comparison of business models with different BW and insect inclusion systems.

## Materials and methods

To fulfill our objective to analyze and compare the viability of different insect-fed broiler supply chains, the deterministic insect production simulation model of Leipertz et al. (2024) was adapted by adding broiler meat production steps and broiler specific input parameters to the simulation model.

### Basic model structure

**Fig. 1:**
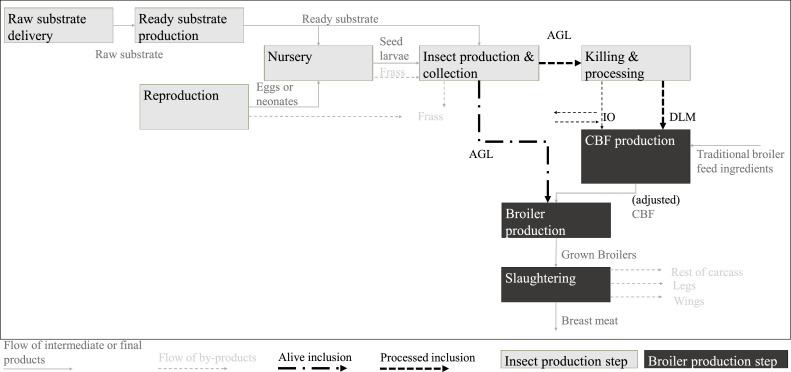
Insect-fed broiler breast meat supply chain structure with alive or processed inclusion: visualization of production steps, intermediate or final products, and by-products in the supply chain.

Fig. 1 displays the structure of the adapted supply chain simulation model to produce insect-fed broiler breast meat. In total, the model, contains 9 production steps from “raw substrate delivery” and “reproduction” of insects up to “slaughtering” of broilers. Each production step yields a product (e.g. neonates) or transforms intermediate outputs (e.g. seed larvae or grown broilers) in final products (e.g. breast meat).

The basis for the insect production steps (light grey marked) is the economic simulation model of Leipertz et al. (2024). The “reproduction”, which encompasses all life stages of the flies (larvae, adults and eggs), provides eggs or neonates (newly hatched larvae) to the consecutive step. The “raw substrate delivery” delivers raw substrates to the “ready substrate production”, which processes them into a mixture with the right texture, moisture and nutritiousness to feed the larvae. The “nursery”, which is the first and 5 days long feeding step, is followed by the “production and collection” of the larvae, which is the second and 6 days long feeding step. The resulting alive grown larvae (**AGL**) can be killed and processed in the “killing and processing” step to obtain defatted larvae meal (**DLM**).

Valuable by-products that occur along the insect production chain are frass (mixture of insect feces and substrate) in the “production and collection” step and insect oil (**IO**) in the “killing and processing” step.

The first broiler breast meat production step (dark grey marked) is the “CBF production”. In case of *processed inclusion* (thick regular dashed lines) the “CBF production” step uses DLM and IO together with traditional broiler feed ingredients to produce the intermediate product: CBF. The model assumes that the supply and demand of DLM exactly match within the supply chain. Opposed to that, IO can be sold or bought as by-product such that ratios of DLM and IO in broiler feed match. In case of *alive inclusion* (thick regular dashed lines), the “killing and processing” step is by-passed, AGL are directly transferred to the “broiler production” step and the “CBF production” step uses traditional feed ingredients to produce an adjusted CBF.

Finally, the “broiler production” step produces broilers that are forwarded to the “slaughtering” step which has broiler breast meat as main output. Rest of carcass, legs and wings are treated as valuable by-products.

### Simulation model adaption

The first added production step is the “CBF production” step. It is based on example diets in the two broiler feeding studies from Dörper et al. (2024) (slow growing broilers) and Souza Vilela et al. (2021) (fast growing broilers). A weighted average was used to summarize the feeding phases. Different oils were treated as one, because the nutritional difference is minor and for simplicity reasons chemically produced amino acids were also summarized. Meat and bone meal, which was included in the fast growing feed study, was split up into soybean meal and oil according to its ingredients to fit the Dutch circumstances.

In case of *processed inclusion*, the goal was that the protein and the fat content that originate from simulated insect products resemble the insect-originated protein and fat of the example diets. For the fast growing feeding study, it was necessary to re-calculate the insect-originated protein contents of the example diets by using a nitrogen-to-protein conversion factor of 4.67 (Janssen et al., 2017). Afterwards, DLM inclusion levels were slightly adjusted to eliminate differences in the protein contents. Resulting insect-originated fat differences were compensated by IO inclusion adjustments.

In case of *alive inclusion*, an exact match of insect-originated protein and fat in modelled and example died was not possible because protein and fat are contained in a fixed ratio in AGL. Thus, AGL inclusion was fixed to exact 5% and 10% of DM content. Resulting differences in protein and fat content compared to the example diet were compensated by a mix of soybean meal and oil.

Average broiler feed formulations were used to calculate percentage changes of the ingredients in CBF with insect inclusion compared to no insect inclusion. The prices of traditional CBF (without insect products) for the two BW systems were retrieved from Wageningen Livestock Research (2023) and taken as a benchmark. To calculate the CBF prices that include insect products, the benchmark prices were adjusted by ingredient percentage changes and respective ingredient prices. More detailed insights in the composition and prices of assumed CBFs can be found in Appendix I.

Costs for transportation, processing and margin are implicitly part of the CBF price.

The second production step that needed to be added was the “broiler production” step. The structure of the “broiler production” module was retrieved from Gocsik et al. (2013) and updated with new data from Wageningen Livestock Research (2023) and van Horne (2020). Full costs were modelled, consisting of feed, buildings and inventory, labor, purchase of day old chicken, electricity, heating, water, interest of bound capital in animals, healthcare, litter, enrichments, catching, manure disposal, and general costs.

Lastly, the “slaughtering” step had to be added. This step respects different cut-up part percentages for the two BW systems BLS and conventional. Costs in the “slaughtering” step are modelled with lump sums given in van Horne (2020).

### Input parameters

The parameterization of the insect production steps is extensively described in Leipertz et al. (2024). The parameterization of the broiler production steps was mainly done by existing literature. Commodity prices were based on price lists of feed suppliers (Nuscience) and stock market prices.

Table 1 contains the inputs that were used as result of the parameterization process. Some inputs, like the price for traditional poultry feed, are different in the two BW systems. The traditional poultry feed for conventional broilers costs 390 €/tWM and thus is 15 € (390 €–375 €) more expensive than BLS traditional poultry feed. Prices for calcium carbonate (54 €/tWM) up to common oil (971 €/tWM) are the same in both BW systems and used to adjust the traditional poultry feed prices like described above.

**Table 1:** Input parameters for the three production steps: CBF production, broiler production, and slaughtering

BW system	Conventional	Both	BLS	Source
**CBF production**				
Traditional poultry feed (€/tWM)	390		375	
Calcium carbonate (€/tWM)		54		Personal communication^1^
Monocalcium phosphate (€/tWM)		620		Personal communication^1^
Salt (€/tWM)		130		Personal communication^1^
Natriumbicarbonat (€/tWM)		480		Personal communication^1^
Sunflower rot (€/tWM)		261		Personal communication^1^
Chemically produced amino acids (€/tWM)		2000		Personal communication^1^
Wheat (€/tWM)		269		Commodity prices^2^
Soybean meal (€/tWM)		406		Commodity prices^2^
Common oil (€/tWM)		971		Commodity prices^2^
				
**Broiler production**				
General parameters				
Growth period (d)	41		56	
Feed conversion rate (kg DM feed/kg life weight)	1.40		1.80	
Mortality (%)	3.50%		2.50%	
Purchase price for young chicken (€/chicken)	0.35		0.385	
Labor				
Full time labor equivalent (#)		1		(size determination)
Own labor price (€/h)		25		Authors expertise
Building and inventory				
Maximum stocking density (kg/m²)	42		25	
Investments in barn (€/m^2^)		303		
Investments in inventory of barn (€/m^2^)		124		
Investments in covered veranda (€/m^2^)	-		263	
				
**Slaughtering**				
Cutup percentages				
Carcass yield (% of live weight)	73.1%		71.4%	van Horne
Breast meat (% of live weight)	22.2%		19.2%	
Wings (% of live weight	8.7%		8.7%	
Legs (% of live weight)	22.7%		22.9%	
Cutup prices				
Wings (€/kg)				
Legs (€/kg)				
Rest of carcass (€/kg)				
Lumpsums				
Slaughtering (€/kg carcass weight)				
Deboning (€/broiler)				
Costs of disposal of poultry waste (€/kg waste)				
				van Horne
				
				
Breast meat (% of live weight)				
Wings (% of live weight				
Legs (% of live weight)				
Cutup prices				
Wings (€/kg)		1.50		
Legs (€/kg)		1.00		
Rest of carcass (€/kg)		0.30		
Lumpsums				
Slaughtering (€/kg carcass weight)		0.330		
Deboning (€/broiler)		0.130		van Horne
Costs of disposal of poultry waste (€/kg waste)		0.005		

Abbreviations: BLS, better life one star; BW, broiler welfare; CBF, commercial broiler feed; tWM, ton wet matter.

1 J. van Harn (Wageningen University, Wageningen, The Netherlands, personal communication): Price list of Nuscience, September 2023

2 Average based on monthly data between Sep.2018 and Sep.2023 retrieved from the data gathering portal IndexMundi

The slow growing broilers in the BLS system have 15 days longer time up to slaughter weight than broilers in the conventional system (56 days vs. 41 days). Consequently, the DM based feed conversion rate in the BLS system (1.80 kg DM feed/kg life weight) is higher than in the conventional system (1.40 kg DM feed/kg life weight). In contrast, the mortality in the BLS system (2.5%) is improved compared to the conventional system (3.5%). However, young chickens are a bit more expensive in the BLS system. Following the approach of van Horne (2020) the broiler farm size is determined by the work that can be done by 1 full time labor equivalent which is paid 25 €/h in both BW systems. Building and inventory requirement is higher in the BLS system due to lower maximum stocking density (25 vs. 42 kg/m²).

Coming to the parameterization of the “slaughtering” step, the cutup percentages slightly differ between the two BW systems. Most importantly, the fast-growing broilers have 3 percentage points more breast meat (22.2 vs 19.2 % of live weight). Again, following publication of van Horne (2020), prices for wings (1.5 €/kg), legs (1 €/kg), and rest of carcass (0.3 €/kg) are fixed for both BW systems and differences in production costs are only imposed on the breast meat. Slaughtering lumpsums are the slaughtering (0.33 €/kg carcass weight), deboning (0.13 €/broiler), and costs of poultry waste disposal (0.005 €/kg waste).

### Analysis approach

In this section, the main production setups are defined and the analysis of their default situation as well as their situation under specific input parameter settings is described.

The default situation of the production setups is analyzed by presenting the most likely amounts and costs in insect-fed broiler breast meat production, by describing the least possible cost increase of broiler breast meat, and by conducting a breakeven analysis.

The situation under specific input parameter settings is analyzed with a breakeven analysis and a sensitivity analysis.

**Fig. 2:**
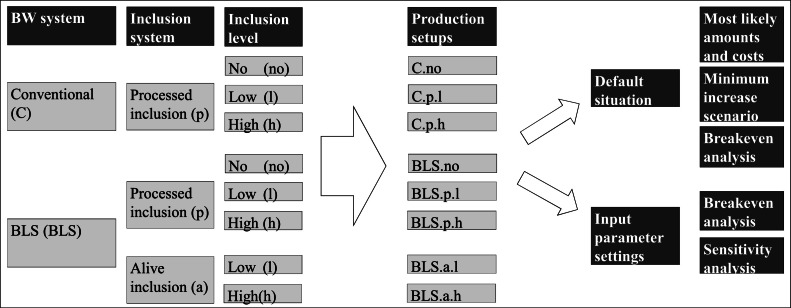
Combination of BW systems, inclusion systems and inclusion levels into production setups and applied analyses of their default situation and input parameter settings.

***Production Setups.***Fig. 2 shows the different production setups that were used. The setups are based upon two BW systems, Conventional (C) and BLS (BLS), combined with two inclusion systems, processed (p) or alive (a) and three inclusion levels: no, low (l; 5%DM insect inclusion in the broiler feed), and high (h; 10%DM insect inclusion in the broiler feed). Due to a lack of data regarding the use of alive inclusion of insects in the conventional BW system, alive inclusion is only applied to the BLS BW system. Consequently, a total of 8 production setups were parameterized. The IDs C.No, C.p.l, C.p.h, BLS.p.l, BLS.p.h, BLS.a.l, and BLS.a.h were used to categorize the production setups.

***Default Situation.*** For all these production setups, the default situation is the basic situation, using the most likely input parameters presented in the third column of Table 2. For example, the assumed most likely values of protein and fat content in AGL are 37.6% and 20%.

**Table 2:** Most likely input parameter levels and input parameter settings: Best and worst case realistic variations

ID	Description	Most likely	Best case	Worst case	Explanation	Source
	**Common**					
C.1	AGL composition (adjusted CBF adjustable)					
C.1.1	Protein in AGL (CBF adjustable, %DM)	37.6%	42.6%	32.6%	5% points variation	Personal communication^1^
C.1.2	Fat in AGL (CBF adjustable, %DM)	20.0%	30.0%	10.0%	10% points variation	Personal communication^1^
C.2	Change in feed prices					
C.2.1	Soybean meal (€/tWM)	406	546	304	5% & 95% percentile	Commodity prices^2^
C.2.2	Common oil (€/tWM)	971	1,657	628	5% & 95% percentile	Commodity prices^2^^,^^3^
C.2.3.c	Conventional poultry feed (€/tWM)	390	504	317	Min and max variation	Wageningen Livestock Research (2023) & 4
C.2.3.b	BLS poultry feed (€/tWM)	375	485	305		
C.3	Broiler production parameters					
C.3.1	Broiler FCR change	0.0%	-9.7%	2.4%	Min and max	
C.3.2	Broiler mortality change	0.0%	-38.8%	123.1%	Min and max	& Souza Vilela et al. (2021)
C.3.3.c	Breast yield (% of live weight)	22.2%	23.2%	21.2%	1% points variation	
C.3.3.b	Breast yield (% of live weight)	19.2%	20.2%	18.2%	1% points variation	
	Insect production scale (qualitative)	Medium,	Large,	Small,		
C.4	equals: Substrate amount (tWM substrate/yr)	60,434,	181,301,	20,053,	Interview	Leipertz et al.
	equals: DLM price (€/tDM)	5,209	4,364	5,987		
	**Alive inclusion**					
A.1	AGL composition (adjusted CBF fixed)					
A.1.1	Protein in AGL (CBF fixed, %DM)	37.6%		32.6%	5% points decrease	Personal communication^1^
A.1.2	Fat in AGL (CBF fixed, %DM)	20.0%		10.0%	10% points decrease	Personal communication^1^
A.2	Feeding equipment and labor					
A.2.1	Equipment (€/m² net barn)	124		137	10% increase	Wageningen Livestock Research (2023)
A.2.2.c	Labor (conventional, h/1000 animal places)	3.73		4.52	6 min/cycle and 1 min/d	van Horne (2020)
A.2.2.b	Labor (BLS, h/1000 animal places)	10.28		11.31		
	Mortality of AGL in storage					
A.3	Mortality (%)	0.0%		4.2%	Appendix	Personal communication^1^

Abbreviations: AGL, alive grown larvae; BLS, better life one star; CBF, commercial broiler feed; FCR, feed conversion rate; tDM, ton dry matter; tWM, ton wet matter.

1 A. Dörper, T. Veldkamp and P. Shah (Wageningen University, Wageningen, The Netherlands, personal communication),

2 Monthly data from Sep. 2018 to Sep. 2023 retrieved from the data gathering portal IndexMundisss

3 Monthly average of palm oil, rapeseed oil and Soybean oil retrieved from the data gathering portal IndexMundi

4 Most likely values taken from Wageningen Livestock Research (2023), and min and max values compared to 5 yr average from Agrimatie (2023) used as variation in %.

***Input Parameter Settings.*** However, the input parameters presented in Table 2 can have considerable variations. Thus, also, the best and worst case variations from the insect inclusion perspective, which are deemed realistic, were used. Furthermore, short explanations for the variations and the sources are given. Whereas the input parameter settings under the heading “Common” are applied to both inclusion systems, input parameter settings under “Alive inclusion” are applied specifically to the alive inclusion system.

Especially, the input parameter settings C.1 and A.1 are characterized by further underlying assumptions. Whereas C.1 variations analyze the impact of AGL composition changes if the CBF can still be adjusted, A.1 variations assume that (adjusted) CBF is already fixed, pelletized and delivered to the poultry farm.

The protein and fat content is influenced by the raw substrates and underlies some variations over the year (Gold et al., 2021). Based on commercial AGL examples in the Insectfeed research consortium, the realistic variation of protein (C.1.1) and fat (C.1.2) content was set to 5 and 10 percentage points. C.2 variations deal with realistic changes in feed and feed ingredient prices. As soybean meal and common inclusion rates differed most between the feed formulations of the production setups, their price variation was included in the analysis.

The feeding studies of Souza Vilela et al. (2021) and Dörper et al. (2024) give different impacts on FCR, breast meat yield and mortalities in broiler production depending on the production setup. The min and max values of these studies were taken as realistic variations in input parameter setting C.3. Finally, the C.4 input parameter setting represents the impact of different insect production sizes.

As indicated above, situation A.1 assumes a fixed adjusted CBF. If protein or fat content of AGL vary, poultry producers have to compensate missing nutrients by increasing the supplied amount of AGL. This may lead to an oversupply of some nutrients.

Situation A.2 handles the possibility that additional feeding equipment and labor might be required in the “broiler production” step in case of alive inclusion. Unfortunately, hard data for this possibility is not available. With regard to the labor, the Insectfeed consortium agreed that per cycle 6 min/1000 broiler and per day 1 min/1000 broiler could be a realistic assumption. Regarding the equipment, a maximum of 10% investment increase was selected to assess the importance.

The last input parameter setting, A.2, is concerned with the storage of AGL. If the AGL are not produced at the poultry farm itself, AGL have to be stored, which can cause mortality in some cases. Besides the most likely value of almost no mortality, a potential average mortality of 4.16% was assumed, which results from 1 delivery per week and an assumed mortality curve which can be found in Appendix II.

***Most Likely Amounts and Costs.*** For the default situation with most likely input parameters, first, the required amounts of intermediate products to produce 1 ton broiler breast meat are described; thereafter, the most likely net cost contribution of the different production steps of the supply chain are provided; that is followed by an analysis of main cost factors, by-product revenues, and transition costs with implications for the most likely price of breast meat in dependence of the production setup.

***Minimum Increase Scenario.*** To estimate the least possible impact on breast meat costs, a minimum increase scenario was introduced which assumes (opposed to the default assumption) that broiler by-products, legs, wings, and rest of carcass, undergo the same cost increase in percent as the main product, breast meat.

***Breakeven Analysis.*** For all 8 production setups, in the default situation as well as in the situation under input parameter settings, the breakeven analysis calculates required breakeven prices of raw substrates, frass, AGL, or DLM such that insect-fed broiler breast meat becomes price competitive with breast meat without insect product inclusion.

The first step was to calculate a reference price for breast meat that has to be adhered. The reference price is the broiler breast meat price, within the respective BW system (conventional or BLS), without insect product inclusion, but including potential changes due to varying input parameter settings.

Afterwards, the MS Excel solver is used to test, individually, how much the prices of raw substrates, frass, AGL, or DLM have to change such that the simulated broiler breast meat price is equal to the respective reference price.

***Sensitivity Analysis.*** Finally, the sensitivity analysis estimates the impact of the best and worst case variations of input parameter settings (Table 2) on the currently prevailing costs. Special focus lies on the impact of variations on the cost difference between insect-fed broiler breast meat and broiler breast meat without insect inclusion. Therefore, the difference between the most likely price difference and the price difference in input parameter settings is displayed.

## Results

### Default situation

***Required Amounts of Intermediate Products.*** All values in Table 3 are standardized on 1 ton broiler breast meat. Specifically that means to produce 1 ton broiler breast meat in the C.No system, 6.30 tDM CBF and 1,877 grown broilers are required. Within the conventional BW system (C.No, C.p.l, and C.p.h), the amount of CBF and number of broilers is similar because no impacts on FCR and cutup parts due to insect inclusions were included in the default situation. The same holds true for the BLS BW system. However, the BLS BW system has a higher basic requirement for grown broilers (2,170 # broiler) and CBF (9.3 tDM) per t breast meat.

**Table 3:** Amounts required to produce 1t broiler breast meat for different production setups

BW system		Conv.	Conv.	Conv.	BLS	BLS	BLS	BLS	BLS
Inclusion system			Processed	Processed		Processed	Processed	Alive	Alive
Inclusion level		No	Low	High	No	Low	High	Low	High
ID		C.No	C.p.l	C.p.h	BLS.No	BLS.p.l	BLS.p.h	BLS.a.l	BLS.a.h
Raw substrate	tDM		1.24	2.49		1.92	3.83	2.11	4.22
Ready substrate	tDM		1.23	2.47		1.90	3.79	2.09	4.18
Neonates	units of 100,000		59.56	119.59		91.84	183.48	101.14	202.28
Seed larvae	tDM		0.02	0.05		0.04	0.08	0.04	0.08
AGL	tDM		0.27	0.55		0.42	0.84	0.47	0.93
DLM	tDM		0.22	0.45		0.34	0.69		
CBF	tDM	6.30	6.30	6.30	9.30	9.30	9.30	8.84	8.37
Broiler	#	1,877	1,877	1,877	2,170	2,170	2,170	2,170	2,170
Breast meat	t	1	1	1	1	1	1	1	1

Abbreviations: AGL, alive grown larvae; BLS, better life one star; BW, broiler welfare; CBF, commercial broiler feed; DLM, defatted larvae meal; tDM, ton dry matter.

The (adjusted) CBF requirement is a little lower in BLS and alive inclusion (8.84 and 8.37 tDM CBF) because AGL are provided separately from pelletized CBF.

Within the same inclusion levels, the requirements for intermediate insect products, DLM up to raw substrate, are always higher for the BLS BW system compared to the conventional BW system. To produce 1 ton BLS breast meat with high and processed inclusion (BLS.p.h), 0.69 tDM DLM, 0.84 tDM AGL, 183 units of neonates (18,3 Mio individuals), and 3.83 tDM raw substrate are required.

Using 4.22 t raw substrate and 202 units of neonates, the required amounts of intermediate insect products are even a bit higher for the alive inclusion with high inclusion level and BLS BW system (BLS.a.h)^2^. Assuming a Dutch breast meat production of ca. 200,000 t completely fulfilled by BLS.a.h, would mean a demand of 2,15 Mio tWM raw substrate (30%DM) and ca. 4 trillion neonates.

Net Cost Contribution of the Different Production Steps. Table 4 presents the net added production costs per production step for 1 ton broiler breast meat. For the C.No setup, the “CBF production” step adds 2,769 € to the net added production costs, followed by the “broiler production” step which adds 1,715€, and finally “slaughtering” reduces the net added production costs by 537€^3^. In total, the net added production costs for 1 ton breast meat in the C.No setups are 3,947 €/t breast meat. As similar cut-up part percentages are assumed within the BW systems, “slaughtering” always reduces net added production costs of broiler breast meat by 537 € in the conventional system and 678 € in the BLS system.

**Table 4:** Net added production costs per production step for different production setups (in €/t breast meat)

BW system	Conv.	Conv.	Conv.	BLS	BLS	BLS	BLS	BLS
Inclusion system		Processed	Processed		Processed	Processed	Alive	Alive
Inclusion level	No	Low	High	No	Low	High	Low	High
ID	C.No	C.p.l	C.p.h	BLS.No	BLS.p.l	BLS.p.h	BLS.a.l	BLS.a.h
Raw substrate delivery		436	874		671	1,342	740	1,479
Ready substrate production		93	187		144	287	158	316
Reproduction		75	151		116	232	128	256
Nursery		106	214		164	328	181	361
Production and collection		184	369		283	566	318	637
Killing and processing		146	294	3,919	226	451		
CBF production	2,769	2,650	2,549	3,251	3,705	3,494	3,548	3,177
Broiler production	1,715	1,717	1,719	-678	3,255	3,259	3,252	3,255
Slaughtering	-537	-537	-537	3,919	-678	-678	-678	-678
Sum (and net production cost per t breast meat)	3,947	4,871	5,820	6,493	7,887	9,280	7,647	8,804

Abbreviations: BLS, better life one star; BW, broiler welfare; CBF, commercial broiler feed.

Looking at all production setups, highest costs are added by “broiler production”, “CBF production”, and “raw substrate delivery”. Whereas “CBF production” and “raw substrate delivery” costs differ for all main production setups, “broiler production” costs are very similar within the BW system. In alignment to the amounts, costs are always higher for the high inclusion level compared to the low inclusion level if everything else is similar.

Although amounts and thus costs in alive inclusion are higher up to the “production and collection” step, the missing “killing and processing” step saves up to 451 €/t breast meat (BLS,p,h) and leads to a lower sum of net production costs compared to processed inclusion (7,647 vs. 7,887 and 8,804 vs 9,280 €/t breast meat).

Most Likely Breast Meat Costs: Cost Factors, By-Product Revenues and Transition Costs. In Table 5 the production costs^4^ are split up into cost factors. The cost factors themselves are separated into three categories: insect production related, broiler meat production related, and common. The lower part of Table 5 displays the by-product revenues and transition costs for the whole supply chain. Finally, total breast meat costs and relative cost increases are provided.

**Table 5:** Cost factors, by-product revenues and transition costs of (insect-fed) poultry supply chains in €/t breast meat

BW system	Conv.	Conv.	Conv.	BLS	BLS	BLS	BLS	BLS
Inclusion system		Processed	Processed		Processed	Processed	Alive	Alive
Inclusion leve	No	Low	High	No	Low	High	Low	High
**Specific insect production cost factors**								
Insect breeding feed		3	7		5	10	6	11
Raw substrate		433	869		667	1,333	735	1,470
Detergent		9	19		14	29	15	30
**Specific broiler meat production cost factors**								
Traditional broiler feed ingredients	2,769	2,571	2,389	3,919	3,532	3,148	3,548	3,177
IO buying^1^		78	157		171	342		
Day old chickens	681	681	681	857	857	857	857	857
Other costs in broiler production^2^	260	260	260	372	372	372	372	372
Slaughtering Lumpsum	1,337	1,337	1,337	1,517	1,517	1,517	1,517	1,517
								
**Common cost factors**								
Labor	178	362	548	565	848	1,131	825	1,085
Electricity	41	132	224	77	216	356	223	369
Gas for heating	72	137	201	154	253	352	175	196
Water	15	35	56	22	53	85	47	75
Storage	0	8	16	0	12	24	16	32
Interest animals	7	9	11	13	17	21	17	20
Building and inventory	460	787	1,115	1,192	1,695	2,197	1,573	1,955
*Sum of production costs*	*5,821*	*6,842*	*7,889*	*8,687*	*10,231*	*11,774*	*9,924*	*11,164*
Frass		49	98		75	151	83	166
IO selling^1^		48	97		75	149		
Wings	588	588	588	680	680	680	680	680
								
Legs	1,023	1,023	1,023	1,193	1,193	1,193	1,193	1,193
Rest of carcass	264	264	264	322	322	322	322	322
*Sum of by-product revenues*	*1,874*	*1,971*	*2,069*	*2,194*	*2,344*	*2,494*	*2,277*	*2,361*
*Sum of transition costs*		*124*	*249*		*191*	*383*	*216*	*431*
Total breast meat costs	3,947	4,995	6,069	6,493	8,078	9,662	7,863	9,235
Increase (%)		26.5%	53.7%		24.4%	48.8%	21.1%	42.2%
Total breast meat costs (min scenario)	3,932	4,640	5,365	6,493	7,678	8,862	7,517	8,542
Increase (%)		18.0%	36.4%		18.2%	36.5%	15.8%	
31.6%								

Abbreviations: BLS, better life one star; BW, broiler welfare; IO, insect oil.

1 In our simulation: more IO has to be bought than appears as by-product in the supply chain.

2 Other costs in broiler production contain: healthcare, litter, enrichments, catching, manure disposal, and general costs.

For the C.No setup, the cost factors traditional broiler feed ingredients (2,769 €) and slaughtering lumpsum (1,337 €) are the most important cost factors. Comparing the C.No and C.p.h setups, costs for traditional broiler feed ingredients slightly decrease (2,769 € vs. 2.389 €) and slaughtering costs are unaffected (1,337 €). However, building and inventory (460 vs. 1,115€) and (insect production) raw substrate costs (0 vs. 869 €) extremely rise in importance.

Also, the medium cost contributors, labor and energy (electricity + gas), extremely increase with insect inclusion. In case of C.p.h vs. C.No, labor costs increase by 208% (548 vs 178 €/t breast meat) and energy costs by 275% (425 vs. 113). Similar trends can be found comparing BLS.No with BLS.p.h or BLS.a.h.

Coming to the by-product revenues, legs are the most valuable by-product, followed by wings and the rest of carcass. However, these are all slaughtering by-products and similar within the BW systems. The most valuable insect production by-product, frass, reduces the broiler breast meat maximum 166 €/t breast meat.

The transition costs stem from Leipertz et al. (2024) and are based on 1 central company conducting all steps. Resulting transportation costs, which can go up to 268 €/t breast meat, mostly represent the transportation of raw substrates to the insect-factory. The margin, which can go up to 170 €/t breast meat, represents a 5% margin on the cost of insect products AGL or DLM. No margin is assumed on the breast meat^5^.

The total broiler breast meat costs (and thus the producer prices) are always higher if insect products are included. The maximum increase is from 3,947 (C.No) to 6,069 €/t (C.p.h) in the conventional system and from 6,493 (BLS.No) to 9,662 €/t (BLS.p.h) in the BLS system. Although in absolute terms smaller, the relative cost increase in the conventional system is a bit more severe (53.7 vs. 48.8%). The comparison of alive and processed inclusion within the BLS system shows that the alive inclusion cost increase is less severe in absolute (9,235 or 9,662 deduced by 6,493) and relative terms (42.2% or 48.8%). However, differences due to BW systems and insect inclusion systems are minor compared to the differences due to inclusion levels. Thus, BLS.a.l increases relative costs only by 21.1%.

Surprisingly, broiler breast meat in the conventional BW system with processed and high inclusion (C.p.h) is almost as expensive as broiler breast meat in the BLS BW system with no insect inclusion (6,069 vs. 6,493 €/broiler).

Minimum Increase Scenario. In the minimum increase scenario, where the price increases are evenly distributed to all cutup parts, relative increases for broiler breast meat are much lower. Moreover, the order of highest costs is changed. The cost increase for BLS.p.h is now the highest (36.5%), directly followed by C.p.h with only 0.1 percentage points difference (36.4%) and by BLS.a.h with 4.9 percentage points difference (31.6%). The lowest relative cost increase has again BLS.a.l with 15.8%.

### Breakeven analysis

Table 6 presents the breakeven prices of raw substrate mix, frass, AGL, and DLM such that the broiler meat prices with insect inclusions, resemble the reference prices (no insect inclusion) in the default situation and respective input parameter settings (Table 2). The reference prices mostly resemble the most likely broiler meat prices without insect inclusion (Table 5). Only high soybean meal and common oil prices can increase the breast meat price without insect inclusion (and therefore the reference prices) by maximum 804 €/t breast meat (7,297 vs. 6,493).

**Table 6:** Breakeven prices of raw substrate mix, frass, AGL, and DLM such that broiler meat price with insect inclusion resembles the reference price (no insect inclusion) in different input parameter settings

		Reference prices	Breakeven prices			
			Raw substrate mix	Frass	AGL	DLM
Conventional	Default situation	3,947	-405	1,913	-166	490
Processed	Best case variations:					
High	High protein content of AGL (C.1.1)	3,947	-397	1,894	-129	539
	High fat content of AGL (C.1.2)	3,947	-342	1,769	-3	546
	High soybean meal price (C.2.1)	4,192	-386	1,868	-76	606
	High common oil price (C.2.2)	4,182	-372	1,838	-16	522
	Best broiler FCR change (C.3.1)	3,947	-292	1,658	347	1,150
	Best broiler mortality (C.3.2)	3,947	-395	1,890	-120	550
	Best breast meat yield (C.3.3)	3,947	-343	1,772	118	856
	Large scale insect production (C.4)	3,947	-261	1,586	14	490
BLS	Default situation	6,493	-384	1,864	-67	618
Processed						
High	Best case variations:					
	High protein content of AGL (C.1.1)	6,493	-372	1,837	-14	687
	High fat content of AGL (C.1.2)	6,493	-319	1,719	99	697
	High soybean meal price (C.2.1)	6,767	-358	1,806	48	766
	High common oil price (C.2.2)	7,024	-320	1,721	220	826
	Best broiler FCR change (C.3.1)	6,493	-279	1,628	406	1,226
	Best broiler mortality (C.3.2)	6,493	-377	1,848	-36	657
	Best breast meat yield (C.3.3)	6,493	-304	1,683	296	1,085
	Large scale insect production (C.4)	6,493	-239	1,537	113	618
BLS	Default situation	6,493	-214	1,482	801	
Alive						
High	Best case variations:					
	High protein content of AGL (C.1.1)	6,493	-204	1,459	848	
	High fat content of AGL (C.1.2)	6,493	-165	1,371	899	
	High soybean meal price (C.2.1)	6,767	-193	1,433	903	
	High common oil price (C.2.2)	7,024	-123	1,276	1,235	
	Best broiler FCR change (C.3.1)	6,493	-120	1,268	1,252	
	Best broiler mortality (C.3.2)	6,493	-208	1,468	830	
	Best breast meat yield (C.3.3)	6,493	-142	1,318	1,147	
	Large scale insect production (C.4)	6,493	-109	1,244	801	
						
	Alive inclusion, worst case variations:					
	Low AGL protein cont. (adjusted CBF fixed) (A.1.1)	6,493	-238	1,534	690	
	Low AGL fat content (adjusted CBF fixed) (A.1.2)	6,493	-272	1,611	527	
	Increased broiler feeding equipment costs (A.2.1)	6,493	-224	1,504	753	
	Increased broiler production labor requirement (A.2.2)	6,493	-227	1,510	740	
	Increased mortality of AGL in storage (A.3)	6,493	-221	1,497	768	
						
	Increased equipment, labor and mortality (A.2 & A.3)	6,493	-243	1,547	663	

Abbreviations: AGL, alive grown larvae; BLS, better life one star; DLM, defatted larvae meal.

For the processed inclusion to become cost competitive under default settings in the conventional BW system, the raw substrate mix for feeding insects may cost −405 €/tDM or less, frass may cost 1,913 €/tDM or more, AGL −166 €/tDM or less, and DLM 490 €/tDM or less to be price competitive. Within the default situation, breakeven prices for AGL and DLM increase to −67 €/tDM AGL and 618 €/tDM DLM in case of BLS BW system and processed insect inclusion. The AGL breakeven price turns into a positive value (801 €/tDM AGL) when insects are fed to broilers alive. Assuming a DM% of 34% that would mean 272 €/tWM AGL.

The input parameter settings “best broiler FCR change” (C.3.1) and “best breast meat yield” (C.3.3), which are both production broiler parameters, have the highest impact on breakeven prices. The “best FCR change” can push the DLM breakeven price to 1,226 €/tDM (BLS.p.h) and the AGL breakeven price to 1,252 €/tDM (BLS.a.h), “best breast meat yield” pushes it to 1,085 and 1,147 €/tDM (DLM in BLS.p.h and AGL in BLS.a.h).

Next in line of importance for the breakeven prices are the input prices for soybean meal and common oil. A high common oil price has an especially high impact on the alive inclusion setup and increases the AGL breakeven price to 1,235 €/tDM AGL.

Although, having almost no impact on insect product (AGL and DLM) breakeven prices, the highest impact on raw substrate mix and frass breakeven prices has the input parameter setting “Large scale insect production”. BLS.a.h would become price competitive if the raw substrate mix costed less than −109 €/tDM or frass more than 1,244 €/tDM.

Improved protein and fat content of AGL and improved broiler mortality can increase the broiler product breakeven prices only slightly.

Below the dotted line in Table 6 the results of special alive inclusion settings are presented. The highest detrimental impact on the AGL breakeven price has a low fat content when CBF is fixed (527 vs. 801 €/tDM) followed by a low protein content when CBF is fixed (690 vs. 801 €/tDM). Increased equipment costs, labor requirement and mortality of AGL in storage all have a small effect on AGL breakeven price. However, they can altogether decrease the AGL breakeven price by 138 (663 vs. 801) €/tDM.

### Sensitivity analysis

**Fig. 3:**
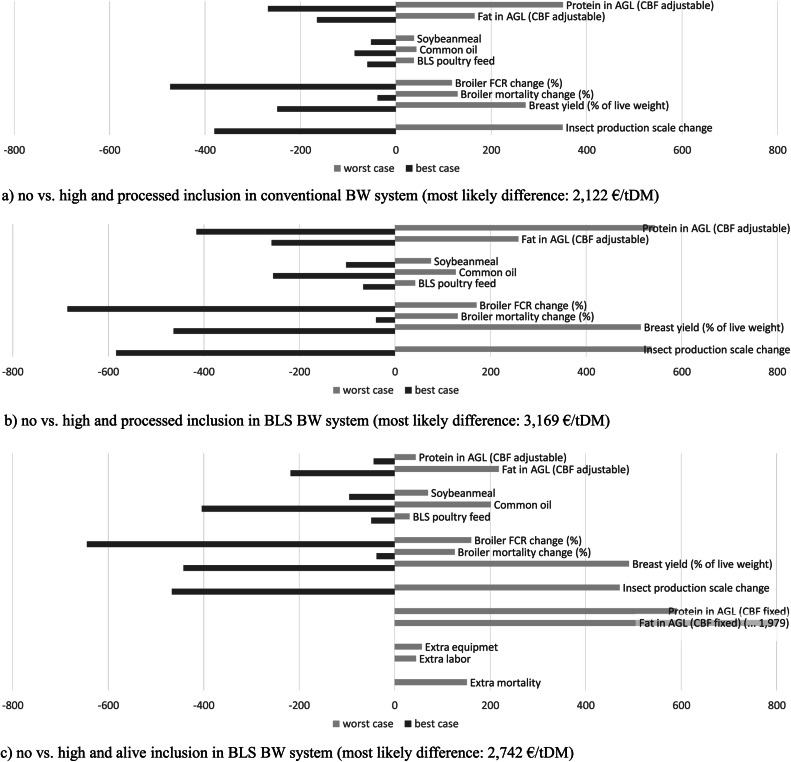
Changes in broiler breast meat price difference between no-insect inclusion and insect inclusion: Most likely price difference (zero point) compared to price difference in best and worst case input parameter settings (in €/t breast meat).

This sub-section focuses on the impact of input parameter settings on the currently prevailing price differences. The zero point of the x-axis in Fig. 3 represents the most likely price difference between insect-fed broiler meat and broiler meat without insect inclusion for the three production setups C.p.h (2,122 €/t), BLS.p.h (3,169 €/t), and BLS.a.h (2,742 €/t)^6^. Dark grey bars show the impact of best case input parameter settings and light grey bars the impact of worst case input parameter settings.

As the same input parameter settings are used, the focus is the result comparison with the breakeven analysis. Broiler FCR change and breast meat percentage change also have one of the highest impacts in the sensitivity analysis. The best case FCR variation seems to have a higher (positive) impact than the threat from the worst case FCR variation.

Opposed to the breakeven analysis, soybean meal and common oil prices only have a medium high impact on the current price difference, because they make up only for a small part of the total costs.

Furthermore, whereas protein and fat content of AGL was slightly less important in the breakeven analysis, especially in case of processed insect inclusion (C.p.h and BLS.p.h), fat and protein content of AGL have a high impact on the current price difference, as it leads to lower inclusion levels of the very costly insect products. Opposed to the processed inclusion, the fat content in the alive inclusion has a very low impact on the current price difference, because it does not impact the inclusion level (fixed to 5 or 10 %DM in the broiler feed).

Whereas, the insect production scale (by definition) has no impact on the insect product breakeven prices, it has a major impact on the current price difference.

The results of the AGL settings in the sensitivity analysis all verify the findings of the breakeven analysis.

## Discussion

The aim of this study was to analyze the economic feasibility of insect product inclusion in the Dutch broiler breast meat production with regard to different production setups.

The focus on Dutch circumstances includes relatively high input prices for labor, energy, and raw substrates which are lower in other countries.

Also, the authors are aware that the inclusion of two different feeding studies and the two different approaches to calculate inclusion levels in processed and alive inclusion might have somewhat influenced the comparability of the results. However, although the calculation of the feed composition was not in favor for the alive inclusion in BLS broiler feed (Appendix III), alive inclusion in BLS broiler feed was still the one with the least relative cost increase. Even if there are some uncertainties, to our knowledge the presented data is the best that is currently available.

None of the insect inclusion production setups were price competitive with no insect inclusion in the default situation. Compared to the cost differences due to insect inclusion levels, the cost differences due to BW and insect inclusion system were minor. To become price competitive in BLS broiler production the DLM (14% fat; 46% protein) and AGL (20% fat, 37.6% protein) may only cost 618 and 801 €/tDM instead of 3,503 and 5,116 €/tDM (Leipertz et al., 2024). These results are in agreement with Tavares et al. (2022) who calculate the economic viability of Tenebrio molitor inclusion in broiler diets with an breakeven price of R$ 4.53/kg (ca. 850 €/tWM) for the insect meal. Although they used a different insect species and an (in default) improved broiler performance, the calculated breakeven price has a similar magnitude as our breakeven prices. As mostly soybean meal and soybean oil are substituted, breakeven prices are anchored to the value of soybean meal and oil.

Although IPIFF, a network of insect producers, expects that ca. 10% of insect production will go into broiler feed in 2030, current costs of insect products seem too high to fulfill that goal. Most consumers accept insects in feed but some are averse towards insect inclusion due to food neophobia, disgust and uncertainties about safety and health (Pakseresht et al., 2023; Szendrő et al., 2020) Therefore, it seems very unlikely that consumers are willing to pay 40 to 50 % more for insect-fed broiler breast meat.

To set this into further context, conventional broiler breast meat with ca. 10% processed insect product inclusion is almost as expensive as BLS broiler breast meat without insect inclusion. Furthermore, BLS broilers fed with ca. 10% processed insect products (4.76 €/broiler) are almost as expensive as organic broilers (5.8 €/broiler: 2.42 €/kg live weight * 2.4 kg/broiler) (van Horne, 2020). The current costs prevent the emergence of intermediate product market with high insect inclusion between the three main BW systems (conventional, BLS, organic) in the Netherlands.

Moreover, the hypothetical demand of 2,15 Mio tWM raw substrate (30%DM) for the total Dutch broiler production will be difficult to procure. Bastein et al. (2013) state that there are in total 8.3 Mio tWM food waste from the Dutch food industry, which is the main (allowed) source for BSF rearing raw substates. However, most of that is already used as feed (Gold et al., 2021). Thus, taking out 2.15 Mio tWM for insect production only to feed broilers will be hardly possible and further increase the prices.

Although including insect products in broiler feed is not price competitive with default assumptions, there are factors that can positively influence the competitiveness. The most impactful parameters were the broiler production parameters, FCR and breast meat yield. They doubled the breakeven prices with assumed best case variations of −9.7 % and +1 percentage point. Various studies describe that antimicrobial peptides (AMP), chitin and lauric acid could improve broiler health and performance (Belhadj Slimen et al., 2023). However, the selected 9.7% decreased FCR has to be seen as the very maximum. It is based on a quadratic formula^7^, which was found by Souza Vilela et al. (2021), extrapolated for their maximum inclusion level (20%). Also, Dörper et al. (2024) found a significantly lower (monthly) FCR for broilers fed with insect products. However at much lower levels: The BLS.p.h production setup reduced the cumulated FCR^8^ by 2.37% and BLS.a.h reduced it by 4.19% (Anna Dörper, unpublished data). A reason for the better FCR in case of alive inclusion could be the improved antimicrobial effect of unprocessed AMP, chitin and lauric acid (Belhadj Slimen et al., 2023; Dörper et al., 2024).

Nevertheless, the calculated AGL breakeven price in (very) best case FCR variation (1,252 €/tDM AGL) is still much lower (−64.3%) than the AGL price given in Leipertz et al. (2024) (3,503 €/tDM AGL).

Although, there was variation in breast meat yield, Dörper et al. (2024) and Altmann et al. (2018) found no significant improvement of breast meat yield in case of insect inclusion. Therefore, the breast meat yield will probably not be a major factor in improving the breakeven prices for insect products.

The second most influential parameters (for the breakeven prices) were increased prices of substituted products common oil and soybean meal. These are external factors which can be an opportunity or a risk at the same time.

Putting everything together, the authors do not expect insects in the Dutch mass poultry feed market in short future, unless important parameters change.

Looking at the breakeven prices or other circumstances that have to be met to enter the Dutch poultry feed market, two different alternatives could be of interest for the long term: Sanitation of negative value substrates, and usage as a feed additive for BW.

The best case input parameter setting (large scale incest production) and production setup (BLS.a.h) resulted a breakeven price of −109 €/tDM raw substrate mix. Although currently not allowed, one potential raw substrate with a negative input price would be pig or cattle manure. Leenstra et al. (2019) report 50 to 250 €/tDM (5 to 25 €/tWM; ca. 10%DM) manure disposal costs for Dutch farmers with an amount of 6.2 Mio tDM cattle and 1 Mio tDM pig manure. Moreover, Agrimatie (2023), the database of Wageningen Economic Research, reports 80.7 €/tDM (ca. 10%DM) manure removal costs for cattle and 172,2 €/tDM (ca. 10%DM) for pig in 2022. These removal costs are expected to further increase, as the Netherlands exemption clause for farms with more than 80% grassland to be allowed to spread 230 kg N/ha manure on sandy soils will gradually abolished to 170 kg N/ha in 2026. Our calculated breakeven price of −109 €/tDM seems to be feasible to reach with manure as raw substrate for insects. Moreover, getting enough manure will probably not be a problem. However, feeding only manure to insects will influence their composition, mortality and developing time. Furthermore, high water percentage of manure may require a mixture with other dry substrates to obtain a product that can be fed to insects. Impacts on the profitability of the supply chain should be tested with the presented model.

At the same time it has to be insured that fed substrates impose no risk to the food chain (Hoffmans et al., 2024). Current legislation only allows high quality substrates of vegetal origin with only a few exemptions (European Union, 2016; IPIFF, Brussels, Belgium, personal communication). That means that the risk of contamination is low. However, discussions are held to use low value substrates in insect rearing. These low value substrates may contain toxins and pathogens, have a higher perishability, vary in nutrient content, and cause reduced consumer acceptability. These risks have to be managed by the supply chain, and the costs associated with this risk management need to be taken into account in the cost price.

The second possibility is the usage as a feed additive for BW. Potentially, improved broiler health due to AMPs, lauric acid, chitin, and (in case of alive inclusion) natural broiler picking behavior might convince some consumers to pay a price premium (Dörper et al., 2024). As low and alive insect inclusion in the BLS BW system imposes the least relative cost increase (21.1%), that should be the main target for this business model. Introduced as a premium niche market, it might be possible to also get a price premium for the other cut-up parts and thus reducing the relative cost increase to 15.8%. A similar business model is operated by Protix with layers, who sells the eggs under the label “Oerei”. Inclusion levels even lower than 5% and improved broiler FCR may help to keep the extra costs of insect inclusion low.

However, the alive inclusion is especially vulnerable to AGL content changes when CBF is fixed. Additional labor, equipment, and mortality only needs to be considered when all appear at the same time. As AGL composition highly depends on the raw substrate used (Gold et al., 2021), constant raw substrate type and quality needs to be ensured, information systems have to be implemented to keep all partners updated on the expected AGL composition, and contracts have to manage or spread the risk, which would be borne completely by the poultry farmers otherwise. Poultry producers, which are a key stakeholder in the insect-fed broiler meat supply chain, will step out of the supply chain if the risk is too high for them otherwise (Saatkamp et al., 2022).

Regarding mortality it was assumed that dead larvae are not provided to broilers because it is not allowed to feed dead larvae. However, in practice they might not be sorted out. Nevertheless, Dörper et al. (2024) conclude that the fat content of the AGL could reduce in storage. Poultry producers will have to balance the costs of AGL delivery frequency against the costs of mortality and fat reduction.

Concerning extra labor and equipment to feed AGL to broilers, lumpsums, which should be seen as the maximum increase possible, were used because there was no other data available. Equipment suppliers like Big Dutchman in cooperation with FarmInsect are already working on practical solutions. The authors do not believe that extra labor and feeding equipment in the broiler barn will be a major hurdle to introducing low and alive insect inclusion in the BLS BW system as a premium niche market.

This study was aimed at the Dutch situation, while the use of insects for feed is evaluated for many other situations as well (Lin et al., 2023). Boarding the scope to a global perspective, for insects to become part of the mass poultry nutrition, insect product prices always have to be in the range of local protein substitute prices. Improved broiler performance may increase the required breakeven prices a bit, but cannot loosen the price anchor. Lower labor and energy costs in Eastern Europe, China or Africa may reduce the insect production costs (J. W. Heesakkers, Bühler, Wageningen, The Netherlands, personal communication). However, most important for viable business models is the local availability of suitable low value substrates in huge amounts (J. Kiem, InsectoCycle, Wageningen, The Netherlands). Legal differences in allowed substrates do have a huge impact on the cost competitive inclusion of insect products in global broiler feed.

## Conclusion

To conclude, in the Netherlands, currently, no insect inclusion is price competitive within same BW system. Higher insect inclusion levels almost linearly increase the costs form 6,493 (BLS.No) to 9.662 (BLS.p.h) €/t breast meat (48.8% increase). Furthermore, introducing a 10% inclusion of insect products in total Dutch broiler production would mean a very high demand for raw substrates (2.15 Mio. tWM) which cannot be sourced only from food waste of food industry.

The highest impact on boiler meat costs have the broiler production parameters, FCR and broiler breast meat yield. However, it is not enough to become price competitive.

Thus, in short run, insect products will not be part of Dutch mass broiler feed.

For the long run, two possibilities for viable business models that include BSF as feed for Dutch broilers are (1) sanitation of negative value substrates, or (2) the usage in a niche market for BW with low inclusion levels of AGL.

Both business models come with increased risks that have to be managed. Especially the alive inclusion comes along with the thread of varying AGL composition when CBF is fixed. The risk, which is mainly beard by the broiler farmer, requires special focus when setting up the supply chain.

## Funding

This work was financially supported by the Dutch Research Council [NWA.1160.18.144]. The Dutch Research Council was not involved in the research process itself.

## Declaration of interest

The authors declare the following financial interests/personal relationships which may be considered as potential competing interests: Mark Leipertz reports financial support was provided by The Dutch Research Council (NWO). If there are other authors, they declare that they have no known competing financial interests or personal relationships that could have appeared to influence the work reported in this paper.

## Figures and Tables

**Table:** 

BW system Inclusion system Inclusion level ID	Conv. No C.No	Conv. Processed Low C.p.l	Conv. Processed High C.p.h	BLS No BLS.No	BLS Processed Low BLS.p.l	BLS Processed High BLS.p.h	BLS Alive Low BLS.a.l	BLS Alive High BLS.a.h
**CBF production** Composition DLM (% of DM) IO (% of DM) Mix of traditional feed ingredient (% of DM) Prices DLM (€/tDM) IO (€/tDM) Mix of traditional feed ingredients (€/tDM)	100.0% 439	3.5% 1.3% 95.2% 5,209 971 429	7.1% 2.6% 90.3% 5,209 971 420	100.0% 421	3.7% 1.9% 94.4% 5,209 971 402	7.4% 3.8% 88.8% 5,209 971 381	100% 423	100% 422
(adjusted) CBF price (€/tDM) Increase (%)	439	605 37.7%	775 76.4%	421	591 40.3%	761 80.6%	423 0.3%	422 0.1%
**AGL inclusion** Composition Adjusted CBF (% of DM) AGL (% of DM) Prices AGL (€/tDM)							95.0% 5.0% 3,742	90.0% 10.0% 3,742
Price of total broiler feed mix (€/tDM) Increase (%)	439	605 37.7%	775 76.4%	421	591 40.3%	761 80.6%	589 39.7%	754 78.9%

*Abbreviations:* AGL, alive grown larvae; BW, broiler welfare; CBF, commercial broiler feed; DLM, defatted larvae meal; IO, insect oil; tDM, ton dry matter; tWM, ton wet matter.

**Table:** 

BW system Inclusion system Inclusion level	Conv. No	Conv. Processed Low	Conv. Processed High	BLS No	BLS Processed Low	BLS Processed High	BLS Alive Low	BLS Alive High
Traditional feed ingredients (€/broiler) DLM (€/broiler) IO (€/broiler) AGL (€/broiler) Buildings, inventory, and land (€/broiler) Labor own (€/broiler) Other costs (€/broiler)	1.48 0.25 0.10 0.57	1.37 0.62 0.04 0.25 0.10 0.57	1.27 1.25 0.08 0.25 0.10 0.58	1.81 0.55 0.26 0.69	1.63 0.83 0.08 0.55 0.26 0.69	1.45 1.65 0.16 0.55 0.26 0.69	1.63 0.80 0.55 0.26 0.69	1.46 1.60 0.55 0.26 0.69
Total broiler costs (€/broiler) Increase (%)	2.39	2.95 23.4%	3.52 47.3%	3.30	4.03 22.1%	4.76 44.2%	3.94 19.1%	4.57 38.2%

*Abbreviations:* AGL, alive grown larvae; BW, broiler welfare; DLM, defatted larvae meal; IO, insect oil.

## APPENDIX

I) Broiler Feed Composition and Prices

**Conventional Broiler Feed Composition:**

*WM% inclusion change of feed ingredients compared to control feed without insect products (Conv. & No)

**BLS Broiler feed composition:**

*WM% inclusion change of feed ingredients compared to control feed without insect products (Conv. & No)

**Price Development of Most Changing Ingredients:**

Main changes in Wheat, Soybean meal, different oils, and poultry feed:

Covid-19 and the Russian invasion made the markets volatile. It is uncertain if the “new plateau” will be stable. Therefore, the 5 yrs averages were taken as most likely prices.

**Calculated Feed Prices in all Production Setups:**

**Calculated Broiler Costs in all Production Setups:**

**Exact tables with all costs (default situation):**

BLS No inclusion

BLS 10 % DLM + IO

II) Assumed Average Mortality Rate when AGL have to be Stored

III) Problem with two different studies

The Table “Calculated Feed Prices in all Production Setups” showed that the DLM inclusion rate is very similar in the two BW systems (C.p.h.: 7.1% vs. BLS.p.h: 7.4%) even tough two different feeding studies were the basis. There is a difference in IO inclusion rates (C.p.h.: 2.6% vs. BLS.p.h: 3.8%), but that is deemed as acceptable because IO is priced as common oil anyway.

Due to the two different approaches to calculate inclusion levels in processed and alive inclusion, the total feed inclusion levels of insect products was a bit higher for BLS.p.h (11.2%) compared to BLS.a.h (10%). However, whereas the insect-originated protein content for BLS.p.h amounted to 3.04% (7.4% * 41.9% protein) of the broiler feed, the insect-originated protein content FSUPPLEfor BLS.a.h is 3.76% (10% * 37.6%). As the alive inclusion is cheaper than the processed inclusion although more insect-originated protein is included in the broiler feed, the conclusion that feeding of AGL to BLS broilers seems to be the most prospective out of the analyzed supply chains can still be hold up.

To obtain results that are 100% comparable, researchers should conduct one feeding study that contains both BW, both inclusion systems, and a comparable amount of insect originated protein. New results could be implemented in the presented model quiet quickly.

IV) Abbreviations

AGL alive grown larvae

BLS Better life one star

BW broiler welfare

CBF commercial broiler feed

DLM defatted larvae meal

IO insect oil

## Footnotes

1 Tavares et al. (2022) calculated total broiler production costs by dividing the total feed costs by 70%. Implicating that fixed costs (remaining 30%) would increase if the feed costs increase.

2 Main reason: inclusion fixed to 5 and 10 %DM and no IO can be bought

3 by-product revenues for legs, wings, and rest of carcass higher than the costs of the slaughtering lumpsums.

4 Net added production costs (Table 4) = production costs – by-product revenues (Table 5)

5 Producer prices are displayed. To get supermarket prices: margin for slaughterhouse and supermarket need to be added. These can vary much and where not available. However, they do not impact the relative cost increases.

6 2,122 = 6,069 - 3,947; 3,169 = 9,662 - 6,493; 2,742 = 9,235 - 6,493

7 FCR (2 to 42 d) = 1.65 - 0.008 * insect product inclusion level (%) - 0.001 * [insect product inclusion level (%) - 10.13]².

8 Dörper et al. (2024) report an average of monthly FCRs. However, the authors got the raw data to calculate the cumulated FCRs, which is the FCR over the whole life span of the broilers.
